# A Microphysiological Cell-Culturing System for Pharmacokinetic Drug Exposure and High-Resolution Imaging of Arrays of 3D Microtissues

**DOI:** 10.3389/fphar.2021.785851

**Published:** 2021-12-21

**Authors:** Christian Lohasz, Jacqueline Loretan, Dario Sterker, Ekkehard Görlach, Kasper Renggli, Paul Argast, Olivier Frey, Marion Wiesmann, Markus Wartmann, Martin Rausch, Andreas Hierlemann

**Affiliations:** ^1^ ETH Zürich, Department of Biosystems Science and Engineering, Basel, Switzerland; ^2^ Novartis Institutes for Biomedical Research, Basel, Switzerland; ^3^ Friedrich Miescher Institute for Biomedical Research, Basel, Switzerland; ^4^ InSphero AG, Schlieren, Switzerland

**Keywords:** pharmacokinetics, microphysiological systems, spheroids, high-resolution imaging, drug testing

## Abstract

Understanding the pharmacokinetic/pharmacodynamic (PK/PD)-relationship of a drug candidate is key to determine effective, yet safe treatment regimens for patients. However, current testing strategies are inefficient in characterizing *in vivo* responses to fluctuating drug concentrations during multi-day treatment cycles. Methods based on animal models are resource-intensive and require time, while traditional *in vitro* cell-culturing methods usually do not provide temporally-resolved information on the effects of *in vivo*–like drug exposure scenarios. To address this issue, we developed a microfluidic system to 1) culture arrays of three-dimensional spheroids *in vitro,* to 2) apply specific dynamic drug exposure profiles, and to 3) *in-situ* analyze spheroid growth and the invoked drug effects in 3D by means of 2-photon microscopy at tissue and single-cell level. Spheroids of fluorescently-labeled T-47D breast cancer cells were monitored under perfusion-culture conditions at short time intervals over three days and exposed to either three 24 h-PK-cycles or a dose-matched constant concentration of the phosphatidylinositol 3-kinase inhibitor BYL719. While the overall efficacy of the two treatment regimens was similar, spheroids exposed to the PK profile displayed cycle-dependent oscillations between regression and regrowth. Spheroids treated with a constant BYL719 concentration regressed at a steady, albeit slower rate. At a single-cell level, the cell density in BYL719-treated spheroids oscillated in a concentration-dependent manner. Our system represents a versatile tool for in-depth preclinical characterization of PK/PD parameters, as it enables an evaluation of drug efficacy and/or toxicity under realistic exposure conditions.

## Introduction

Understanding the relationship between drug exposure (pharmacokinetics; PK) and the biological response (pharmacodynamics; PD) is a key element in pharmaceutical drug discovery (Tuntland et al., 2014). To advance a compound from preclinical to clinical human trials, a wide range of complementary PK/PD characterization studies have to be performed (Tuntland et al., 2014; Li et al., 2019). Despite this thorough preclinical characterization, 9 out of 10 potential drug candidates fail in clinical trials, 75% of which are due to PK/PD-related toxicity or a lack of efficacy (Khalil et al., 2020). These numbers illustrate the need for more predictive preclinical methods that allow for translation of *in vitro* exposure-response-relationships to drug-dosing regimens in patients (Lave et al., 2016).

Current testing strategies for the characterization of PK/PD relationships rely on animal models. However, the preclinical assessment of PK/PD parameters in animal models faces a range of limitations (Prantil-Baun et al., 2018; Ingber, 2020). Animal studies require large numbers of animals to obtain statistically meaningful data, which is laborious, cost-intensive, and ethically questionable (Doke and Dhawale, 2015). Moreover, data that were acquired with animal models do not always translate to humans (de la Torre and Farre, 2004; Cesarovic et al., 2020). Nevertheless, due to the systemic insights that animal models provide, animal-based, preclinical characterization of PK/PD properties of a drug is and will remain a prerequisite to identify effective/safe dosing regimens and to start clinical studies on patients (Prantil-Baun et al., 2018). A major shortcoming of such *in vivo* experiments, are, however, the readout methods, which cannot continuously provide information on the effects of dynamic PK concentration changes in target tissues.

Novel *in vitro* methodologies have gained increasing attention and may help to overcome the shortcomings of *in vivo* experimentation and to improve preclinical evaluation of drug candidates (Cirit and Stokes, 2018). So-called microphysiological systems (MPSs) combine microfluidic technology with advanced, human-based cell-culture models. They feature precise control of the cell-culturing conditions and help to mimic human physiology and disease more closely than traditional *in vitro* methods (Renggli et al., 2019; Sung et al., 2019). Therefore, such systems hold great promise to provide valuable insights into the complex mechanisms underlying the responses of human organs and tissues to potential drug candidates (Marx et al., 2016). As a consequence, they are believed to have the potential to significantly advance pharmaceutical research, to reduce drug development costs and time, and to increase overall dug candidate success rates (Franzen et al., 2019). Some systems already enable the assessment of the effects of dynamic PK exposures of single or multiple drugs on 2D or 3D cell culture models (Lohasz et al., 2019a; McAleer et al., 2019b; Guerrero et al., 2020; Komen et al., 2020). The main limitation of most of these PK exposure systems is, however, that they provide endpoint analysis data, so that short-term and transitory effects of compound concentration changes on the respective target tissues cannot be assessed.

In this article, we present a dedicated microfluidic system that allows for exposure of arrays of 3D multicellular tumor spheroids to dynamically changing PK drug concentrations. At the same time, the system enables continuous monitoring of the resulting effects on spheroid size changes in 3D at short time intervals. The chip system features four individual channels, in each of which up to ten spheroids can be perfused under the same experimental conditions. After characterization of the optical and fluidic properties of the chip system, we exposed spheroids established from fluorescently-labeled T-47D breast cancer cells to three subsequent 24 h-PK concentration profiles of the targeted anti-cancer drug BYL719, a potent inhibitor of the alpha isoform of phosphatidylinositol 3-kinases (PI3Kα). The FDA-approved compound was reported to successfully prolong the progression-free survival among treated breast cancer patients with mutated PI3Kα (Andre et al., 2019). The PK/PD properties of BYL719 have previously been characterized by the periodic measurement of plasma concentrations and tumor size in preclinical studies on rodents (Fritsch et al., 2014) and in clinical trials on patients bearing solid tumors (De Buck et al., 2014). The well-characterized T-47D cell line was selected for this study due to its known sensitivity to BYL719, which manifests as reduced growth upon exposure (Elkabets et al., 2013). Spheroids under exposure to BYL719 were monitored in the microfluidic system at short time intervals by an automated 2-photon microscope with an actively perfused immersion system.

## Materials and Methods

### Microfluidic Device Fabrication

The microfluidic chip system was fabricated by modifying and expanding a commercially available microfluidic cell-culturing chip (Akura™ Flow, InSphero AG, Schlieren, Switzerland). The injection-molded polystyrene chip has the size of a microscopy slide (2.5 mm × 7.5 mm) and features two independent microfluidic channels, each of which interconnects ten microtissue (MT) compartments (Lohasz et al., 2019b). The chip was originally developed for cell-culturing applications using gravity-driven perfusion. For this project, the chip was modified to enable pump-driven liquid perfusion.

Modifications of the chip included the removal of wall structures at the top of the chip, and a widening of specific channel sections at the bottom of the chip by computer numerical controlled (CNC) micro milling. Thereafter, the channel structures at the bottom of the chip were sealed with pressure-sensitive adhesive film (Brooks Life Sciences, Chelmsford, MA, United States) and coated with a hydrophilic, non-adhesive coating, provided by InSphero AG, to prevent attachment of the spheroids during experimentation. All modifications performed to the chip are detailed in Supplementary Figure S1.

For interfacing the modified chips with the pumps, a dedicated aluminum frame was CNC machined. The frame featured recesses for the two microfluidic chips and allowed for connection of the microfluidic chips to commercially available microfluidic tubing and connectors. For compatibility with standard microscopy stages, the assembled aluminum frame featured the same footprint as standard microtiter plates (ANSI/SLAS, 2004).

An acrylic lid was fabricated to tightly seal the access ports to the MT compartments. A layer of polydimethylsiloxane (PDMS) was used as sealing material between the lid and the MT compartments. The lid featured internal channel structures that enabled the local application of a vacuum above of the MT compartments for chip degassing and prevention of bubble formation during experiments. Details of the on-chip degassing approach were described elsewhere (Huang et al., 2020).

Supplementary Methods 1 contains a more detailed description of the chip modifications and the device fabrication.

### Cell Culture and Spheroid Model Generation

The human breast-cancer cell line T-47D (ATCC, Manassas, Virginia, United States) was routinely maintained in growth medium, composed of RPMI-1640 (Bioconcept AG, Allschwil, Switzerland), 2 mM l-Glutamine (Bioconcept AG), 10 mM HEPES (Bioconcept AG), 1 mM sodium pyruvate (Bioconcept AG), 0.02 mg ml^−1^ bovine insulin (Sigma-Aldrich), 1x Penicillin/Streptomycin (P/S, Bioconcept AG), and 10% fetal calf serum (FCS, Bioconcept AG) at 37°C in a humidified 5% CO_2_ incubator. For imaging, RPMI-1640 was replaced with RPMI-1640 without phenol-red (Bioconcept AG). T-47D constitutively expressing mKate2-labeled nuclei were generated by lenti-viral transduction with NucLightRed (Essen BioScience, Ltd., Newark, United Kingdom), followed by selection and maintenance of transduced cells in the presence of puromycin (2 μg ml^−1^). Multicellular spheroid generation was initiated 4 days prior to the start of the *in vitro* PK/PD experiment by seeding 200 cells in 50 μl of growth medium into each well of a non-adherent Akura™ 96 plate (InSphero AG). The seeding density, resulting in spheroids with diameters of 200–250 μm, was based on prior cell-seeding density titrations performed with T-47D cells (data not shown). All cell culturing was performed under standard mammalian cell culturing conditions (37°C, 95% humidity, 5% CO_2_).

### Imaging

**FIGURE 1:**
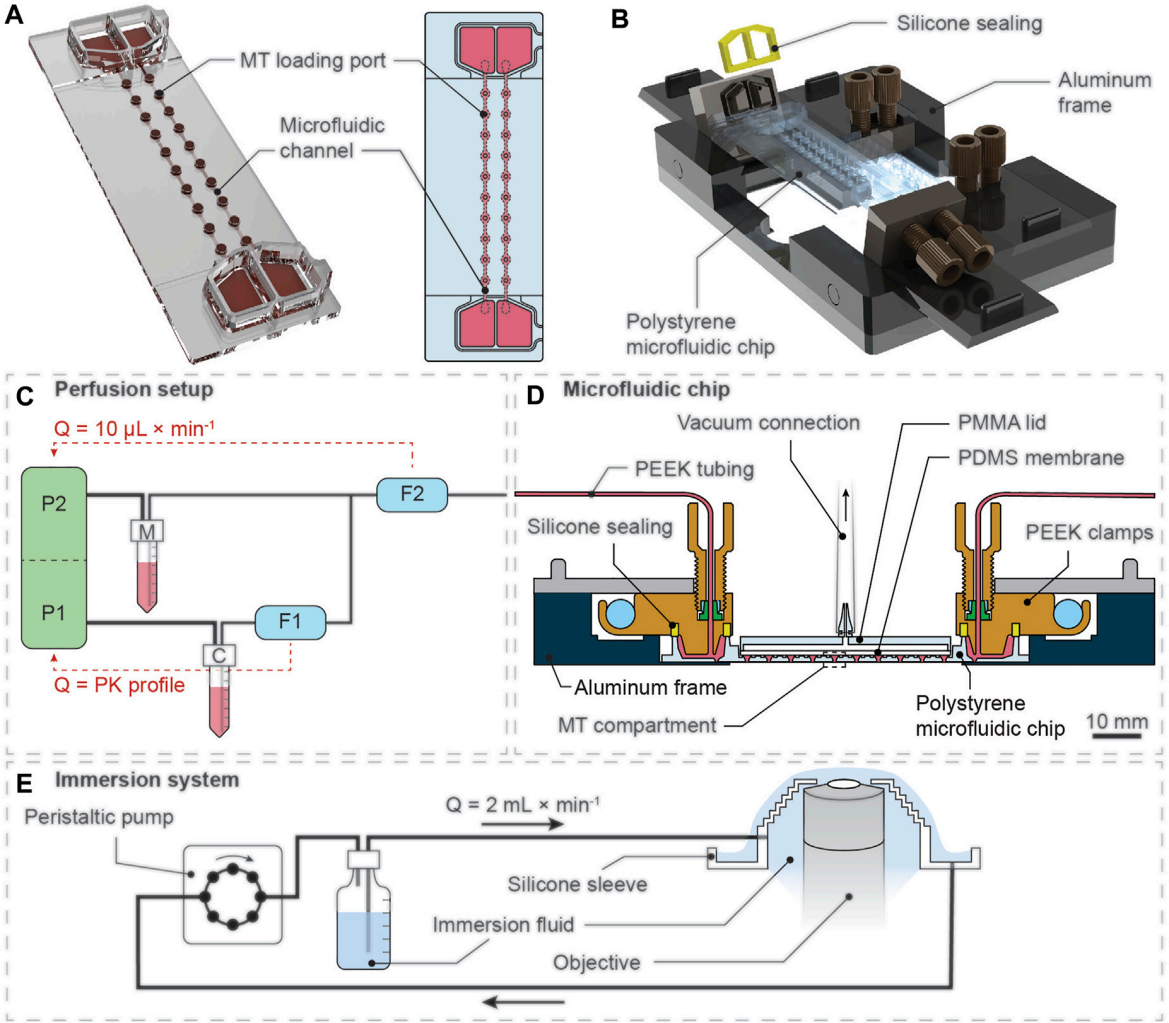
Illustration of the experimental setup for pharmacokinetic (PK) drug exposure of 3D spheroids. **(A)** Rendered 3D representation (left) and top-view schematic drawing (right) of the microfluidic chip with top-open MT loading ports that are located directly on top of the MT compartments along the microfluidic channel. **(B)** Rendered 3D representation of the custom-made aluminum frame that can connect two microfluidics chips to a microfluidic perfusion setup. **(C)** The perfusion setup consisted of two individual pressure pumps (P1 and P2) that were connected to a compound reservoir (C) and a medium reservoir (M). The flow-rate sensor F1 was located upstream of a T-junction, which connected the two liquid lines, and was used to control the drug concentration over time. The flow-rate sensor F2, located downstream of the T-junction, was used to control P2 to maintain a total flow rate (Q) of 10 μL min^−1^. **(D)** The disposable microfluidic chip was clamped into the aluminum frame. The aluminum frame enabled connection of the chip to standard microfluidic tubing. The silicone sealing on top of the reservoirs and the vacuum lid enabled leakage-free operation over extended periods. **(E)** A continuous-flow immersion system, actuated by a peristaltic pump, constantly supplied immersion fluid to the microscope objective to enable automated, long-term imaging at multiple positions of the microfluidic device without manual renewal of immersion fluid.

High-resolution images were recorded on an Olympus FV-MPERS system, equipped with a Newport SpectraPhysics DS+ 2-photon-laser and a stage-top incubator to allow for long-term imaging under mammalian cell-culture conditions. A XLPLN25XSVMP (25x, NA 1.0) water-immersed lens was used for imaging. Fully automated long-term imaging was enabled by a customized fluidic system that constantly replenished the immersion fluid around the lens (Rausch and Peker, 2020). To this end, a peristaltic pump with a set flow rate of 2 ml min^−1^ constantly pumped pre-heated water into a silicone sleeve around the objective. Excessive water dripped into a reservoir at the bottom of the sleeve and was returned to the reservoir bottle by the same peristaltic pump (Figure 1C). The excitation wavelength was set to 800 nm (0.5% laser power) for visualization of the cells using the transmission detector and 1,040 nm (3% laser power) for excitation of mKate2. The spatial resolution was 1 × 1 × 2 µm³. 121 images were acquired along the *Z*-axis. The time interval between successive images varied slightly between the experiments and ranged from 84 min to 94 min.

### Experimental Setup and Operation

For pharmacokinetic drug exposure experiments, a stock solution of BYL719, dissolved in dimethyl sulfoxide (DMSO, Sigma-Aldrich), was added to the cell-culture medium to achieve final concentrations of 50 μM (PK exposure) or 9.3 μM (AUC-matched constant exposure). Vehicle control medium was prepared with final DMSO concentrations matching those used for the exposure profiles (i.e., 0.5% DMSO). One day prior to starting an experiment, vials of cell-culture media with loosely attached lids were placed in a cell culture incubator over night to equilibrate to the experimental conditions and to degas at 37°C.

All liquid-handling and chip-loading steps were performed under sterile conditions. Before starting the experiment, the microfluidic chip was UV-sterilized for at least 30 min in a sterile cell culture work bench. Three hours before starting the experiment, the channels of the chips were filled with 200 µl of degassed cell culture medium. The chips were then incubated at 4°C for at least 2 h to dissolve potential microbubbles at sharp edges and corners of the channel structures. The medium was then replaced twice by fixing the chips at a tilted position and by adding fresh, pre-heated medium to the top reservoirs. The medium flowing into the bottom reservoirs was simultaneously removed. Cell-culture-medium-filled chips were inserted into the dedicated aluminum frame, and the reservoirs were closed on both sides with the PEEK clamps. Previously generated spheroids were manually transferred into the MT compartments of the chip using a multi-channel pipette. The detailed loading procedure was already described elsewhere (Lohasz et al., 2019b). After loading, all spheroids were visually inspected to ensure their integrity. The MT compartments were closed by carefully placing the PDMS sheet onto the access ports, before pressing down with the acrylic lid. The lid was fixed with six screws that were equally tightened using a torque wrench.

The perfusion system (Figure 1A) was actuated and precisely controlled by a set of pressure control units and flow rate sensor units (Fluigent GmbH, Jena, Germany). To run PK dosing protocols, each inlet of the microfluidic chip was connected to two different vials containing 1) plain cell culture medium and 2) medium containing the compound/vehicle. Both vials were individually controlled by separate pressure controllers. Before entering the microfluidic chip, the two medium lines were merged in a T-junction. Two sequentially aligned flow-rate sensor units with direct feedback-loops to the respective pressure control units were used to precisely control the flow rates of both liquids. The first flow rate sensor was located upstream of the T-junction and controlled the flow of the compound/vehicle-containing medium. The second flow rate sensor was downstream of the T-junction and controlled the overall flow into the chip by feeding back to the pressure pump actuating the flow of the plain cell culture medium. The total flow rate of both liquids was maintained at 10 μL min^−1^. The flow rate of 10 μL min^−1^ was chosen based on the following technical considerations: 1) the flow rate was sufficiently high to ensure a high turn-over of medium around the spheroid culturing site and, hence, rapid and precise drug concentration changes, and 2) the applied flow rate allowed for a run time of at least 72 h without disturbances through refilling of the inlet reservoirs. For dosing protocols featuring constant compound/vehicle concentrations, only one vial with the desired compound/vehicle concentration in cell culture medium was connected to the inlet of the microfluidic chip. For all inlet tubing, polyether-ether-ketone (PEEK) tubing with an inner diameter (ID) of 125 µm (Fluigent GmbH) was used. The cell culture media were placed in a heating block (Eppendorf AG, Schoenenbuch, Switzerland) and were maintained at 37°C throughout the entire experiment. The outlet ports of the chips were connected to fluorinated ethylene propylene (FEP) tubing with an ID of 500 µm (Fluigent GmbH), and the effluent was collected in waste bottles. To avoid effects of gravity on the flow rates, the flow path from the medium-containing vials to the effluent waste bottles was setup in a steadily inclining direction (i.e., the end of the medium line was positioned higher than the start of the medium line).

Before connecting the tubing to the perfusion frame, all air was evacuated from the tubing by flushing with cell culture medium at high flow speed. To connect the fully assembled and loaded microfluidic chip system to the medium line, the flow was kept running with plain cell culture medium at a low flow rate of 5 μL min^−1^.

After placing the microfluidic chip into the stage-top incubator of the microscope and connecting it to the perfusion setup, the system was monitored for at least 30 min under experimental perfusion conditions (10 μL min^−1^) without compound dosage to ensure stable and smooth operation. The perfusion protocol with three 24 h-dosing intervals and a total duration of 72 h was initiated thereafter. Simultaneously, the automated imaging protocol was started to continuously record image stacks of all spheroids in the chip over the entire duration of the perfusion protocol.

Between experiments, all reusable parts of the experimental setup was sterilized with ethanol by flushing the PEEK tubing, by submersion of all small components (PEEK clamps, silicone sealing, microfluidic tube connectors and PDMS film), and by spraying the aluminum frame and the PMMA lid.

### Pharmacokinetic Profile Tracking

To optically track and verify the intended PK profile, a microfluidic chip was prepared for experimentation without loading spheroids and was connected to the experimental setup. A stock solution (25 μg ml^−1^) of fluorescein sodium salt (Sigma-Aldrich, Buchs, Switzerland) in water was used as a fluorescent tracer and was added to the compound vial that was connected to the inlet of the chip. The PK perfusion protocol that was used for this experiment started with 1) 100% dye solution for 30 min, followed by 2) a switch to 0% dye solution for 2.5 h to test for the dynamic range of the fluorescence signal and the response time of the system after changing the concentration. Then, the sequence was followed by 3) one 24 h-PK profile intended to be used for spheroid exposure to BYL719. Images of regions within and outside of the microtissue compartment were taken at 10 min intervals.

### Compound Quantification

To verify that the concentrations of BYL719 in the system matched the desired PK profile, a sampling system was attached to the outlet of the experimental system (Supplementary Figure S2). For this verification, an M-switch (Fluigent GmbH) was connected to the chip outlets via FEP tubing (ID = 250 µm). The M-switch enabled to switch between up to ten different downstream flow paths. Eight flow paths were directed to individual wells of a 96-well microtiter plate. PEEK tubing with an ID of 125 µm (Sigma-Aldrich) was used between the M-switch and the 96-well plate to avoid large dead volumes within the tubing. An additional flow path connected the M-switch to a waste bottle.

The switching of the M-switch between the different flow paths was integrated into the PK profile perfusion protocol, so that samples would be directed into the microtiter plate at specific time points. In between the different sampling intervals, the effluent was guided into the waste bottle. Every 24 h, the ends of the sample tubing were manually moved to another set of wells on the microtiter plate by sliding the custom-made lid to the next row.

At the end of the experiment, the samples were stored at -20°C until further quantification of the BYL719 concentration by UPLC/LC-MS/MS as follows. A sample volume of 25 µl was mixed with 25 µl of internal standard (1 μg ml^−1^ NVP-BYZ649; IS) and extracted by addition of 200 µl acetonitrile. After sonication for 5 min, samples were centrifuged to remove precipitated proteins. Supernatants (70 µl) were mixed with 60 µl of HPLC-water prior to analysis of 5 µl aliquots by UPLC/MS-MS. The samples were injected into a ACQUITY UPLC BEH C18 column (Waters™ 1.7 µm particle size, 2.1 × 50 mm), equilibrated with a mobile phase consisting of 95% solvent A (0.1% formic acid in water) and 5% solvent B (0.1% formic acid in acetonitrile), at a flow rate of 600 μL min^−1^. Following a latency period of 15 s, the sample was eluted with a linear gradient, in which solvent B levels were increased from 5–100% over a period of 39 s followed by 81 s at 100% solvent B. The column was prepared for the next sample by re-equilibration during 15 s under starting conditions. The column eluent was directly introduced into the ion source of a triple quadrupole mass spectrometer (Waters Corporation, Milford, MA, United States) controlled by Masslynx™ software. Electrospray positive ionization (ESI +) multiple reaction monitoring was used for the MS/MS detection of the analyte. Precursors to product ion transitions of 442.3 ► 328.2 [m/z] for NVP-BYL719, and 445.2 ► 331.2 [m/z] for (IS) NVP-BYZ649 were used. The limit of quantification (LOQ) for NVP-BYL719 was set to 0.2 ng ml^−1^ (CV and overall bias less than 30%). Regression analysis and further calculations were performed using TargetLynx™ (Micromass) and Excel™ (Microsoft). Concentrations of unknown samples were back-calculated according to the peak area ratios of analyte/IS from a calibration curve constructed using calibration samples spiked in control media.

To correlate the measured values to the applied PK profile, the expected concentration during the sampling interval was calculated by considering 1) the applied average concentration over the sampling duration, and 2) the length of the flow path through the entire system, which included a delay of the concentration change occurrence between the pressure pumps and the M-switch.

### Data Analysis

Before any statistical analysis, the image sequences for each spheroid were inspected visually, and a spheroid was excluded from the analysis if one or both of the following conditions were met: 1) too many images were empty (especially if multiple images including that at the start were missing), 2) the spheroid moved out of the region of observation.

Two distinct image analysis pipelines were set up, one for a 2D projection-based readout and one for a single-cell-based 3D readout. The 2D image analysis comprised three steps: maximum intensity projection, segmentation of the spheroid and statistical summary. All image processing was done using macros for the Fiji platform (Schindelin et al., 2012); the statistical analysis employed the R/RStudio environment (R Core Team, 2013; RStudio Team, 2020). In the first step, the 4D data were reduced by maximum intensity projection along the *z*-axis and stored as both, 3D stacks (xyt) and individual PNG files for each time frame, respectively. The latter served for visual inspection of the spheroid image over the whole time course and helped to decide whether to exclude certain frames or entire spheroids from the statistical summary, as mentioned above. The segmentation of the spheroid projection was accomplished at each time point performing a Gaussian blur (sigma: 1px), morphological external gradient (structuring element (SELM): disk, radius: 1px) and subsequent auto-threshold (method: Mean). Then morphological opening (SELM: disk, radius: 1px), closing (SELM: disk, radius: 3px) and fill holes was applied. The area of the largest remaining object was finally calculated as the measure of spheroid size.

To enable averaging across experiments, the obtained area values were linearly interpolated at regular time intervals with a spacing of 84 min using the “approx” function of the R package. The area values were then normalized to a suitable time point at the start. As for one experiment the area value for t = 0 was not available for all spheroids, the earliest possible time point t = 4 was chosen for normalization. For this reason, the normalized growth curves for the groups “Vehicle constant” and “BYL719 constant 9.3 µM” start at values slightly different from 1.

For the single cell-based 3D analysis we employed the NoviSight™ 3D cell analysis software (Olympus Corporation). First, the spheroid was segmented as a whole by applying a rolling-ball background correction (radius = 50 µm) and Otsu thresholding. Within the obtained spheroid region, the NuclearL module of NoviSight™ was used to segment individual nuclei. This module was parametrized by an expected approximate nucleus size of 7 µm. This analysis provided information on the number, density and center positions of all detectable nuclei. As detailed below, the spheroids could not be observed in their entirety. Therefore, a 30 µm-thick disk-like section half way between the midpoint and the bottom of the spheroids was selected for the subsequent analysis in R/RStudio. Within this section, two regions of interest were defined: the “center” region comprised all nuclei at a maximum distance of 35 µm from the center axis of the spheroid, whereas the “shell” region comprised nuclei with a maximum distance of 35 µm to the spheroid surface. Here, the convex hull of all considered nuclei approximated the spheroid surface. For the nuclei in both regions, the number of nearest neighbors within a maximum radius of 20 µm was obtained using the nn2 function of the R package RANN (Arya et al., 2019). Similar to the 2D projection-based analysis, the number of nearest neighbors was interpolated at regular time intervals of 84 min to enable averaging across experiments.

### Statistical Analysis

The acquired data is generally represented as mean values and their standard deviations (SD). Number of replicates are given as n_e_ and n_s_, indicating the number of independent experiments and the total number of spheroids in these experiments, respectively. Locally weighted scatterplot smoothing (LOWESS) was used to visualize trends in the relative size change of the spheroids and was performed with smoothing windows including 10 data points.

## Results

### Implementation of the Experimental Setup

The perfusion platform for pharmacokinetic drug exposure and real-time monitoring of 3D microtissues was realized by repurposing and adapting an existing, polystyrene-based microfluidic chip system. This chip system was developed for culturing of arrays of 3D MTs under gravity-driven perfusion through repeated tilting (Lohasz et al., 2019b). Real-time optical monitoring of the MTs required the chip system to remain in a horizontal position during the entire experiment. This was achieved by modifying the microfluidic chip by CNC micro milling and by developing a metal frame that enabled connection of the chip liquid ports to pressure pumps.

The modifications of the polystyrene chip included an enlargement of narrow channel regions to decrease the hydraulic resistance in the chip and to prevent clogging of the channels by cell debris. Wall structures at the top of the chip were removed to flatten the chip surface and to enable a tight sealing of the MT loading ports.

The metal frame could accommodate up to two single-use microfluidic chips and enabled fast exchange. The modified microfluidic chip enabled simple and fast MT loading using standard pipetting equipment. Each chip featured 20 MT compartments to have a sufficient number of technical replicates per experiment. The custom-made lid sealed the MT compartments after loading and acted as an on-chip degassing unit during experimentation. Potential air bubbles within the channels were removed through the gas-permeable PDMS layer without interrupting or interfering with the multi-day experiments. At the same time, the PDMS layer retained the liquid medium in the MT compartments and channels.

**FIGURE 2:**
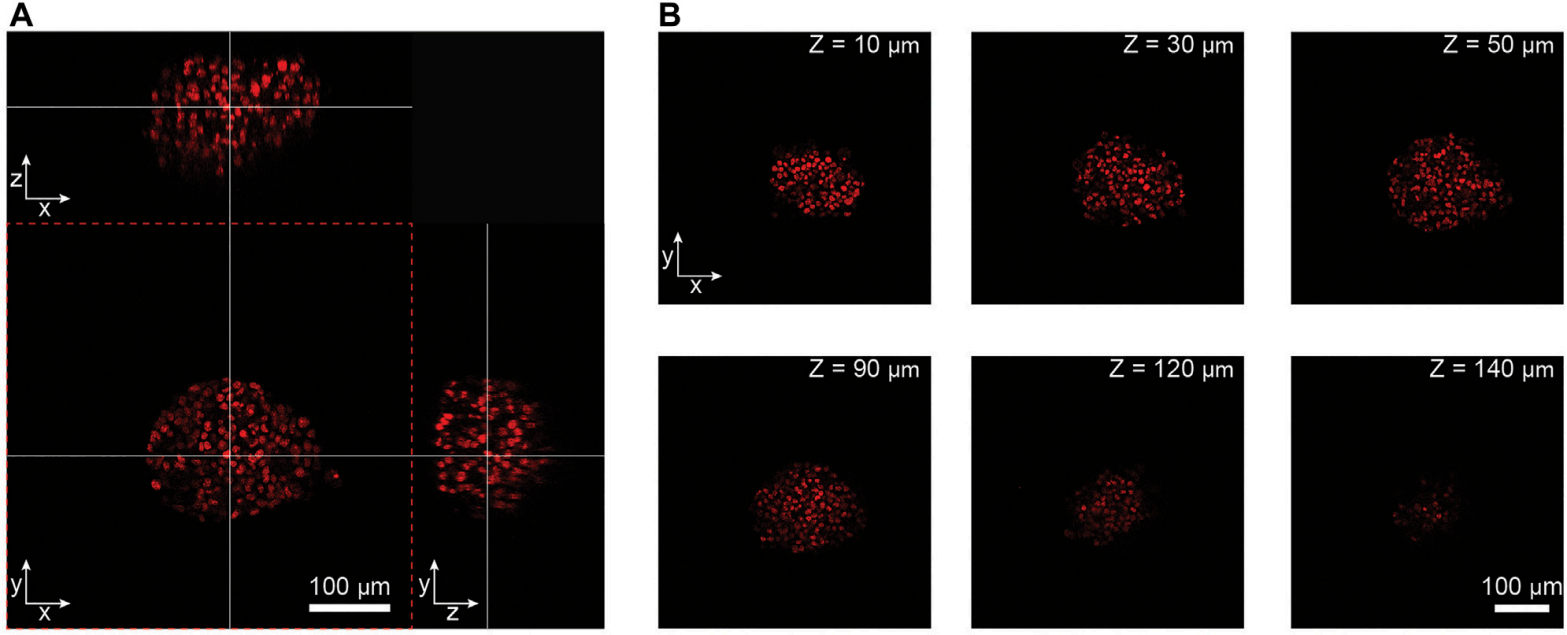
Analysis of imaging penetration depth into NucLight Red-labelled T47-D tumor spheroids. **(A)** Orthogonal views of a spheroid imaged within the microfluidic chip. The white crossing lines indicate the positions of the orthogonal views through the spheroid (scale bar = 100 µm). **(B)** Representative imaging slices of the spheroid at different depths along the *Z*-axis (scale bar = 100 µm).

Two-photon microscopy, combined with an actively perfused immersion system, provided an automated solution to image the spheroids in the chips in 3D at single-cell resolution at minimal photo-toxicity over extended time periods. Figure 2A shows a 3D representation of a NucLight Red-labeled T-47D breast-cancer spheroid within a MT compartment of the chip. Representative images shown in Figure 2B illustrate the optical penetration depth into the biological sample, demonstrating that the 2-photon imaging system allowed for 3D imaging of an entire spheroid of approx. 150 µm diameter at single cell-resolution. The temporal resolution at which the spheroids could be monitored depended on the number of individual spheroids being imaged, and the number of images per Z-stack into the spheroid. For example, in experiments with 20 spheroids and 120 images per Z-stack, the temporal resolution was approx. 85 min. The images of a spheroid in the perfusion system at discrete time points over a 67-h period are shown in Supplementary Figure S3.

### Validation of PK Drug Exposure Profiles

**FIGURE 3:**
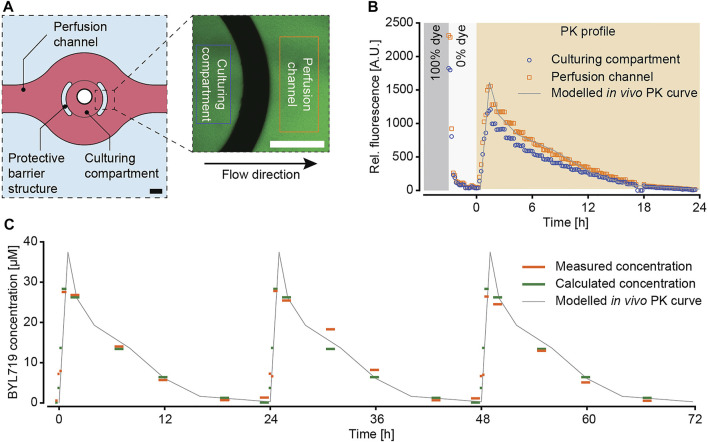
Characterization of the pharmacokinetic (PK) exposure profiles. **(A)** Fluorescein was used as a drug surrogate to track the PK profile by fluorescence imaging. Images were acquired at representative positions of the microtissue culturing compartment and in the perfusion channel. Rectangles in the image illustrate the areas, where the fluorescence intensity was measured (scale bars = 200 µm). **(B)** Evaluation and comparison of the fluorescence signal inside the culturing compartment and in the perfusion channel in the context of the modelled *in vivo* PK curve. The applied concentration profile included an initial sequence that featured 30 min at 100% dye concentration (grey) and 2.5 h at 0% dye concentration (white) before starting the modeled PK profile for BYL719 over 24 h (orange). **(C)** Validation of the modeled PK profile with the anti-cancer compound BYL719 by applying three subsequent dosing cycles with a total duration of 72 h. The applied PK profile (grey) was perfused though the microfluidic chip system. The perfusate was sampled at the outlet of chip and analyzed by mass spectrometry (orange). The calculated concentrations (green) were derived by averaging the different compound concentrations over the sampling period, and by taking the tubing length and the resulting delay of the concentrations between the pressure pumps and the outlet into account.

For our pharmacokinetic drug-exposure studies, we selected the anti-cancer drug BYL719, a phosphoinositide 3-kinase α (PI3Kα) inhibitor that is used to treat certain types of breast cancer. As proof of concept, we aimed to reproduce the *in vivo* PK-profile of daily 50 mg kg^−1^ administrations of BYL719 that had been found to induce clearly perceivable tumor regression in xenografted mice (Fritsch et al., 2014). The corresponding PK profile was implemented using a script to precisely control the pressure pumps of the microfluidic setup. The microfluidic pumping scheme was validated by using the molecule fluorescein as a drug surrogate and by monitoring concentration-dependent fluorescence intensity in the chip over time. The fluorescence intensity was measured within the culturing compartment and in a specific channel region to confirm that the spheroids would be exposed to the intended concentration profile. A schematic drawing of the culturing compartment and a representative image of the intensity measurement are shown in Figure 3. Rectangles mark the regions imaged for intensity measurements. For validation we used a sequence of two subsequent exposure scenarios: in the first part, we switched from a dye concentration of 100% to a concentration of 0% to assess the washout time of a substance; in the second part we used the designed PK profile of BYL719 over 24 h (Figure 3B). The system was operated at a constant overall flow rate of 10 μL min^−1^. Images covering parts of the culturing compartment and the channel region were taken at 10 min intervals.

The analysis of the fluorescence intensity (Figure 3B) showed that the fluorescence signal intensity within the imaging region rapidly dropped after switching from a 100% dye concentration to a 0% dye concentration. After approx. 70 min, the measured relative fluorescence intensity dropped below 5% and remained stable between 2 and 4.5%, which was then considered a complete washout. The recorded fluorescence intensity of the following PK profile of BYL719 precisely reproduced the intended sharp concentration increase within the first 1.5 h, followed by gradually decreasing concentrations over 22.5 h. The measured fluorescence intensity within the culturing compartment was slightly lower than the one in the channel region. This difference was not only observed for the PK profile, but also during the priming sequence with 100% dye solution. A possible reason for this observation may be an imaging artefact due to the different dimensions of channel and MT compartment. Upon normalizing each recorded PK profile to the maximum intensity at 100% dye concentration, both concentration curves were congruent (Supplementary Figure S4). Furthermore, fluid dynamic modeling suggested the concentration change in the medium surrounding the spheroid being complete (more than 90% of target concentration reached) after approx. 10–15 min (Supplementary Figure S5).

In addition to the recorded fluorescence intensity profile, the PK profile was validated by using the compound BYL719 itself. Three cycles of the PK profile were run during 72 h. An automatic sampling unit at the outlet of the microfluidic chip transferred fractions of the perfusate at distinct time points into individual wells of a 96-well plate. The BYL719 concentration in these wells was then analyzed by mass spectrometry (Figure 3C). During the entire PK protocol, the measured BYL719 concentrations correlated well with expected concentrations. These results evidenced 1) the stability of the compound over at least 72 h in cell culture medium at 37°C, and 2) the reliable replication of the modeled BYL719 PK profile in our microfluidic system.

### Tumor Spheroid Response to PK Exposure with BYL719

**FIGURE 4:**
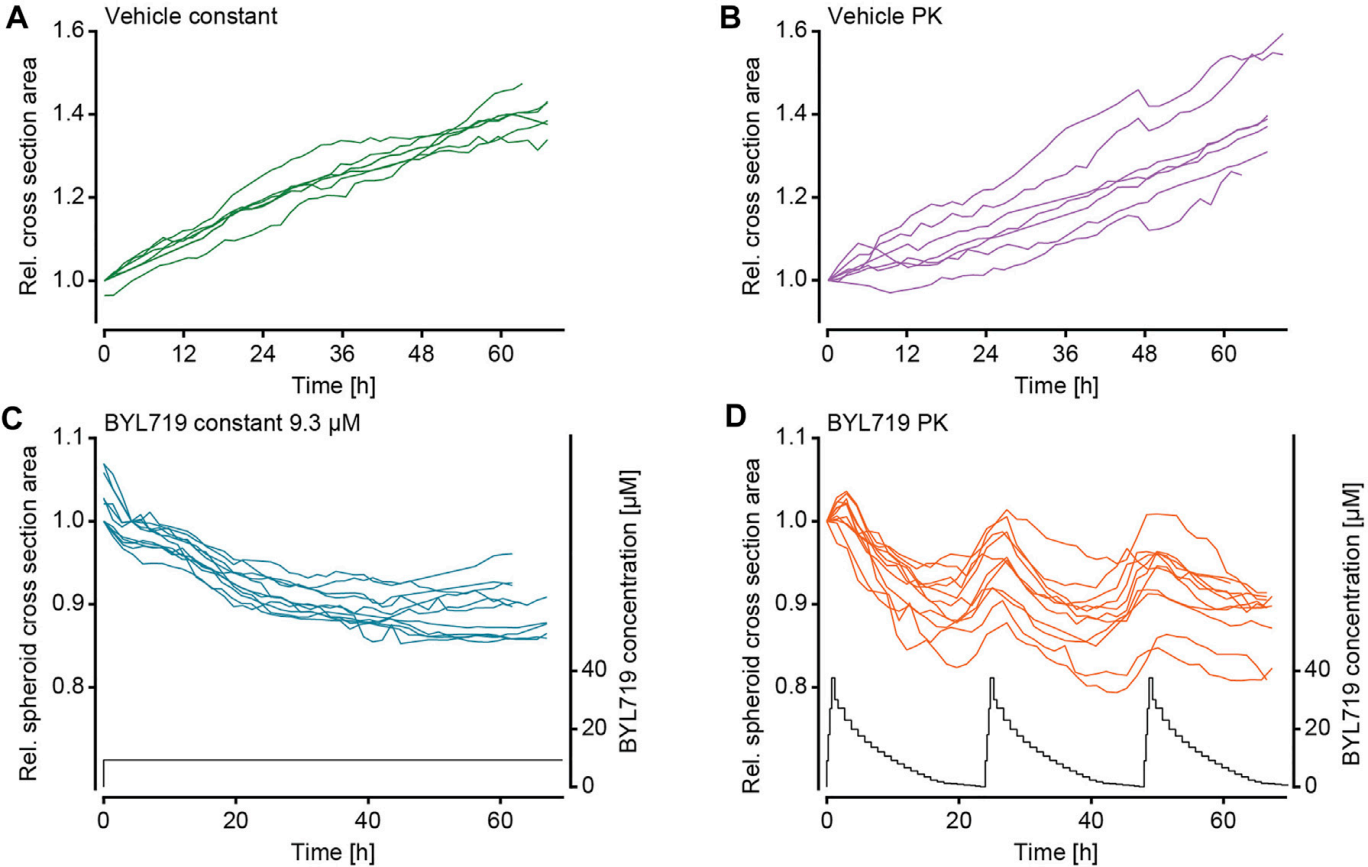
Size changes of NucLight Red-labelled T-47D tumor spheroids under different BYL719 treatment regimens. The relative tumor cross-sectional area of individual spheroids under exposure to **(A)** the constant vehicle control (n_e_ = 2 experiments and n_s_ = 7 spheroids), **(B)** the PK vehicle control (n_e_ = 2 experiments and n_s_ = 7 spheroids), **(C)** constant BYL719 concentration of 9.3 µM (applied concentration shown in black; n_e_ = 2 experiments and n_s_ = 11 spheroids), and **(D)** PK profiles of BYL719 (applied PK profile shown in black; n_e_ = 2 experiments and n_s_ = 11 spheroids).

After validation of the microfluidic system and the pharmacokinetic pumping protocols, the effect of pharmacokinetic BYL719 administration on tumor spheroids was assessed. Scaffold-free breast cancer spheroids consisting of T-47D cells were generated and loaded into the microfluidic chip. To avoid the formation of necrotic cores by exceeding diameter of 500 µm (Vinci et al., 2012), spheroids were used at an initial diameter of 200–250 μm, and they did not exceed 400 µm in diameter for the duration of our experiments. T-47D cells were selected due to their known sensitivity to BYL719, which manifests as reduced growth upon exposure (Elkabets et al., 2013). After loading, the chip was connected to the perfusion setup, and the spheroids were allowed to settle for at least 30 min under perfusion conditions without compound dosing. The perfusion protocol for PK dosing of BYL719 and the imaging protocol were then simultaneously initiated. Two different dosing protocols were applied for BYL719 exposure of tumor spheroids: 1) repeated 24 h-cycles of the modeled PK profile that was previously characterized in the chip system, and 2) a constant steady state concentration of 9.3 µM BYL719. Both dosing regimens featured equal areas under the curve (AUCs). Vehicle control experiments with the corresponding dimethylsulfoxide (DMSO) concentrations were run for both treatment scenarios. Figure 4 displays relative cross section areas of individual tumor spheroids under the different conditions during up to 64 h.

The imaging of the tumor spheroids at short time intervals in the microfluidic chip enabled to precisely track the size of the spheroids over time. Measurements of the maximum cross section area showed that the sets of spheroids responded in a similar way to the respective treatment conditions. The growth characteristics of the spheroids were distinct, and we categorized their responses to the treatment conditions in three groups: 1) Continuous growth was observed for vehicle control experiments and spheroids that were exposed to DMSO either at constant concentration (Figure 4A) or in the form of PK profiles (Figure 4B); 2) steadily decreasing size was observed for spheroids exposed to constant concentrations of BYL719 (Figure 4C); 3) PK dosing of BYL719 caused phases of accelerated size decrease (in comparison to constant dosage) of the spheroids at high BYL719 concentrations and gradual size increase at low BYL719 concentrations (Figure 4D).

**FIGURE 5:**
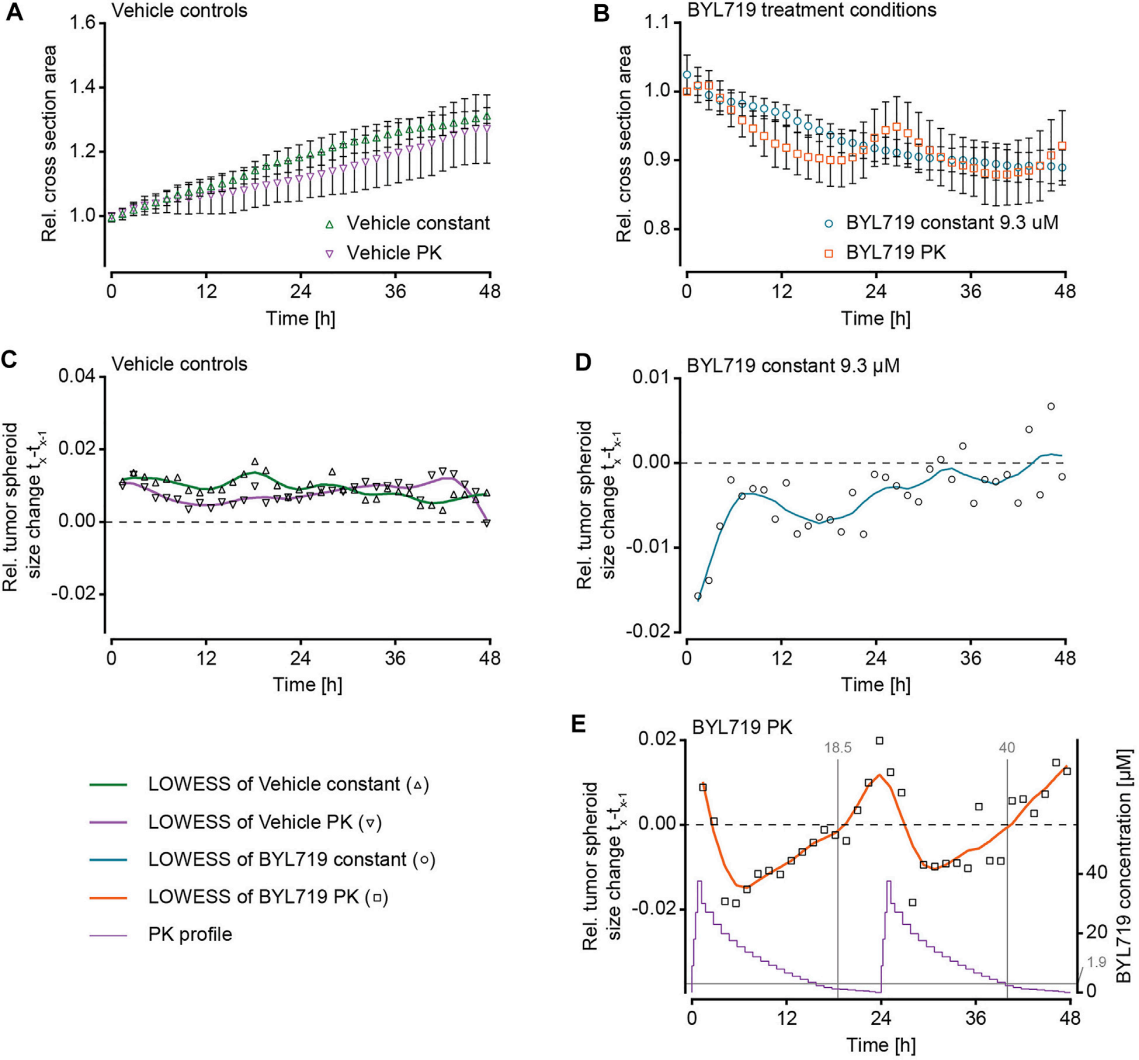
Analysis of NucLight Red-labelled T-47D tumor spheroid size under different BYL719 treatment regimens during 48 h. Relative tumor cross-sectional area of **(A)** vehicle controls under constant exposure or PK exposure to DMSO (Vehicle constant, green triangles; Vehicle PK, purple triangles), and **(B)** treatment conditions under exposure to a constant BYL719 concentration of 9.3 µM (blue circles), or two cycles of a pharmacokinetic (PK) profile of BYL719 (orange squares; data normalized to t = 0 h; data of A- B represented as mean values ±SD; n_e_ = 2 experiments and n_s_ = 7–11 spheroids, as specified below). **(C–E)** The relative size change of the spheroids from one imaging time point (t_x-1_) to the subsequent one (t_x_) under exposure to **(C)** constant and PK Vehicle control (n_e_ = 2 experiments and n_s_ = 7 spheroids per condition), **(D)** constant BYL719 concentrations (n_e_ = 2 experiments and n_s_ = 11 spheroids), or **(E)** PK profiles of BYL719 (n_e_ = 2 experiments and n_s_ = 11 spheroids). The purple line shows the applied PK profile, vertical grey lines indicate the time of transition from negative to positive size change (18.5 and 40 h), and the horizontal grey line indicates the average BYL719 concentration (1.9 µM) at the time of the transitions (data of C – E represented as mean values and locally weighted scatter-plot smoothing (LOWESS) with 10 data points per smoothing window).

As the constant-treatment concentration of 9.3 µM BYL719 was determined as the AUC equivalent of the applied PK profile, a comparison of the overall treatment effects on the growth of the tumor spheroids was only valid after 24-h intervals, as, then, the applied overall dose of BYL719 was the same for both scenarios. A comparison of the spheroid responses to the different treatment conditions was performed after 48 h. Figure 5 shows mean cross section areas of the two vehicle control conditions (Figure 5A) and the two BYL719 treatment conditions (Figure 5B) over 48 h. The constant-concentration and PK vehicle conditions yielded a 1.15-fold or 1.13-fold size increase over 24 h. The overall size increase during the experiment was 1.31-fold under constant DMSO exposure, and 1.27-fold under exposure to PK profiles of DMSO. The size decrease of spheroids upon exposure to constant concentrations of BYL719 was fastest right after starting the treatment, yielding a 0.92-fold decrease within the first 24 h. After these initial 24 h, the curve flattened. The overall size decrease at the end of the experiment was to 0.89-fold. The overall size decrease of spheroids under PK dosing of BYL719 was to 0.92-fold and, thus, similar to the constant-concentration exposure scenario.

The size change of the spheroids was further analyzed by calculating the relative size change between one imaging time point and the next for each of the four treatment scenarios (Figure 5C – E). LOWES smoothing was applied for better visualization of the size change. Analysis of the vehicle-control and constant-concentration exposure groups confirmed the previously described observations: Both vehicle control groups displayed a stable size increase over time (Figure 5C). Constant exposure to 9.3 µM BYL719 resulted in a size decrease with the largest size change at the beginning of the exposure, after which the relative size change decreased and became almost zero at the end of the experiment (Figure 5D). Analysis of the relative size changes of spheroids exposed to BYL719 PK profiles revealed a 24 h-periodicity of interchanging shrinkage and growth of the spheroids (Figure 5E). These periodic patterns corresponded to the 24 h-cycles of the PK profile. A comparison of the relative size changes and the PK profile revealed that the transition from negative to positive size change occurred when compound concentrations in the system were lowest, i.e., towards the end of the PK profile. Switches from positive to negative size changes, on the other hand, immediately occurred after the concentration peaks of the PK profiles.

**FIGURE 6:**
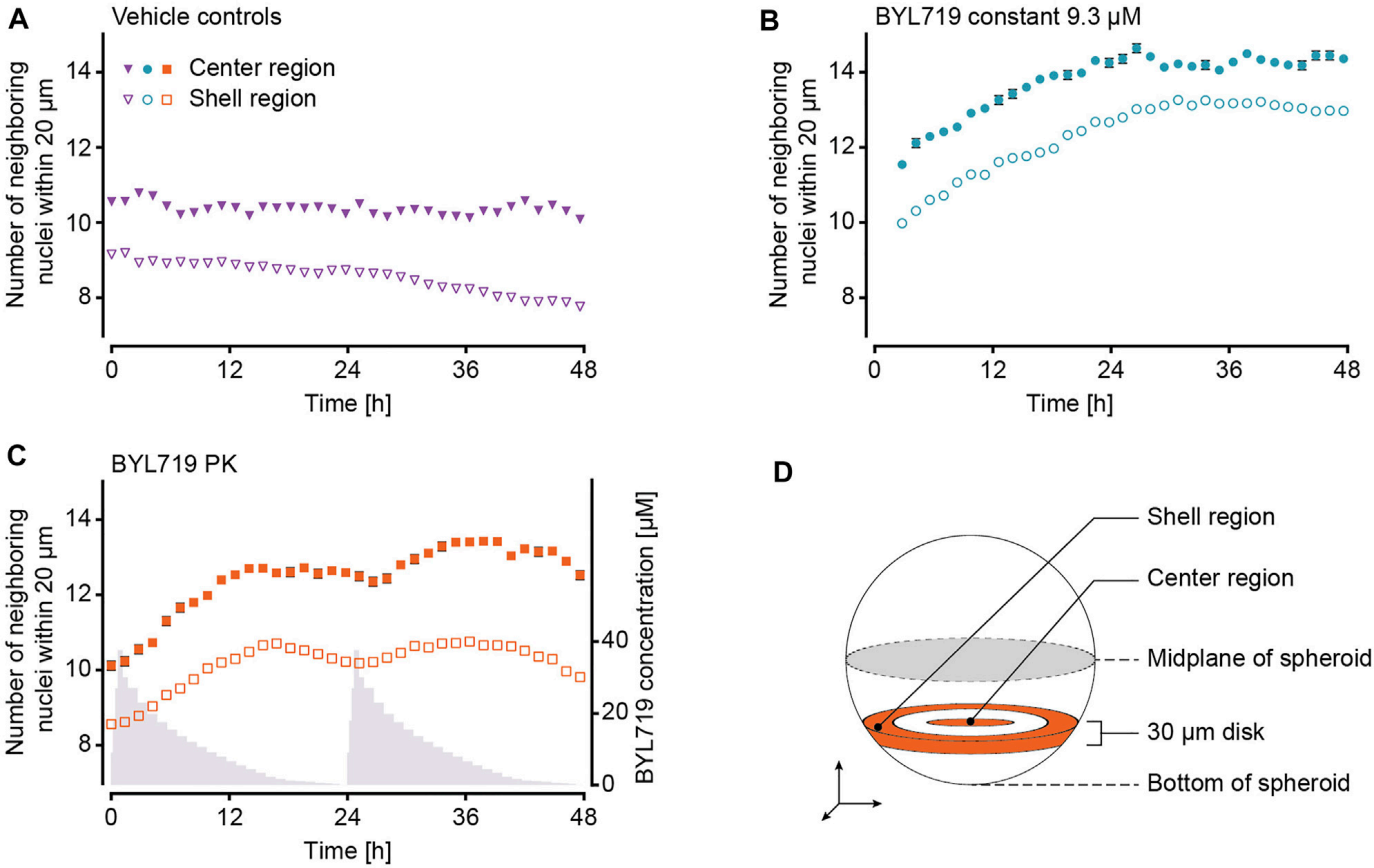
Analysis of the BYL719 effects during 48 h on the number of next neighbors in NucLight Red-labelled T-47D spheroids at single-cell level. The number of nuclei neighboring each cell nucleus within a maximum distance of 20 µm in the center and shell regions of each spheroid under **(A)** constant exposure and PK exposure to DMSO (vehicle controls, n_e_ = 2 experiments and n_s_ = 14 spheroids), **(B)** constant exposure to 9.3 µM BYL719 (n_e_ = 2 experiments and n_s_ = 11 spheroids), and **(C)** PK exposure (in grey) to BYL719 (n_e_ = 2 experiments and n_s_ = 11 spheroids). **(D)** Schematic illustration of the 30 µm-thick disk, within which the center and shell regions were defined. A more detailed description can be found in section 2.7 (Data Analysis).

The use of a multi-photon microscope for image acquisition offered the advantage to evaluate the drug effect at a single-cell level. The NucLight Red labelled nuclei were mapped within a 3D reconstruction of the spheroids. As detailed in Figure 2, not all of the spheroids could be imaged in their entirety, which can ultimately lead to incorrect results when evaluating the total nucleus count per spheroid. Alternatively, we chose the number of nuclei neighboring each cell nucleus within a maximum radius of 20 μm, as an indicator for cell density (Figure 6). This parameter was assessed in a disk-like section of each spheroid featuring a thickness of 30 µm (Figure 6D). The center region was specified as the innermost region with a radius of 35 μm, and the shell region was specified as the outermost 35 µm of each spheroid (see section 2.7). The numbers of neighboring nuclei were generally different in the two specified regions for all treatment conditions, being larger in the center than in the shell region (Figures 6A–C). In control spheroids, the number of neighboring nuclei remained constant in the center, while it decreased in the shell region. Spheroids under constant BYL719 exposure featured increasing numbers of neighbors in the center and the shell regions, with the fastest change in cell density at the start of the experiment. PK exposure of spheroids to BYL719 resulted in increasing and decreasing numbers of next neighbors in the center and shell regions with a 24 h-periodicity.

## Discussion

### Spheroid Exposure and Monitoring at High Precision

We developed a microfluidic microtissue culturing system that enables the exposure of arrays of spheroids to pharmacokinetic compound-concentration profiles, while monitoring them optically in 3D at high resolution. We realized the system by combining 1) a high-precision microfluidic perfusion setup including pressure pumps and flow rate sensors, 2) a microtissue culturing chip in a perfusion frame with on-chip degassing, and 3) a multi-position 2-photon microscope with a fully automated, perfused lens immersion system.

The microfluidic perfusion system enabled flexible programming of concentration profiles to recapitulate compound-specific *in vivo* PK profiles. Tracking of the substance concentration over time with a fluorescent dye and by mass-spectrometric analysis of the perfusate demonstrated that the profiles desired in the chip could be reliably generated (Figure 3). By using fluorescein as a surrogate for the compound, we observed slight differences in the fluorescence intensity measurements between regions in the culturing compartments and the perfusion channel, with lower fluorescence intensities inside the culturing compartment. These differences were detected during the initial dosing of 100% dye solution and throughout the duration of the PK profile. The differences of the measurement values at 100% dye solution and during the PK profile were proportional so that a normalization of the two curves to the initial measurement values at 100% dye solution rendered the PK profiles, measured within the culturing compartment and the perfusion channel, identical. Based on fluid dynamics simulations of the concentration changes within the MT compartment (Supplementary Figure S5), we could attribute the difference in measurement values between the MT compartment and the microfluidic channel to the imaging position. The concentration change inside the MT compartment was slowest in proximity to the downstream barrier structure with respect to the flow direction, which is exactly where all images for the measurements were acquired. Therefore, the selected compartment measurement region was representative for the worst-case scenario of concentration change within the MT compartment, while the concentration was likely to change faster in the rest of the MT compartment. Nevertheless, after 10–15 min, the liquid surrounding the spheroid reached at least 90% of the desired concentration value. Furthermore, the fact that the stepwise concentration decrease during the PK profile was well resolved and clearly visible (Figure 3B) demonstrates the suitability of our system for applying dynamic PK profiles. In both measurement regions, within the MT compartment and within the channel, the concentration steps were similarly sharp. Characterization of the PK profile by quantification of BYL719 in the perfusate of the system showed similar measurement values during all three subsequent dosing cycles at the respective sampling points (Figure 3C). The measured concentrations matched the expected concentrations after correction for the temporal delay between the occurrence of the concentration change in the pumping system and at the outlet of the chip. The fact that PK concentration profiles were reflected in concentration measurements of samples at the chip outlet confirmed that the programmed PK profiles were applied to the entire system and, hence, also to the spheroids within the culturing compartments. These measurements evidenced stability of BYL719 in cell-culture medium at 37°C for at least 72 h. Furthermore, the similarity of the measurements in all three PK profile cycles indicated that ad/absorption of the compound to the chip material or resorption into the medium was minimal, which is in line with the previous characterization of the ad- and absorption properties of the chip system (Lohasz et al., 2019b): The system can, hence, be used for dynamic, long-term compound-exposure scenarios.

The T-47D breast cancer spheroids were formed in purpose-made well plates prior to their transfer and use in the microtissue culturing chip. The off-chip production of spheroids allowed for pre-selection of uniform spheroids (i.e., same shape and size) that were transferred to the chip. This quality-control step increased the reproducibility of experimental results. We repurposed and modified a previously published and commercially available microtissue culturing chip that was originally developed for operation by tilting motion (Lohasz et al., 2019b). The polystyrene-based chip and its microtissue compartments have been proven suitable for the culturing of spherical microtissues in long-term experiments (Kim et al., 2015a; Kim et al., 2015b; Lohasz et al., 2019a; Lohasz et al., 2019b). Modifications to the channel structure and the surface of the chip and the development of a custom-made frame enabled the connection to, and the perfusion with a pressure-driven pumping system. The modular nature facilitated the sterilization of the components of the microfluidic system. Spheroids were loaded into the microfluidic chip in a sterile environment before positioning the chip in the perfusion frame. The closed microfluidic system was then transferred into the stage-top incubator of the imaging setup. Cleaning of the system after experimentation was simple, as the microfluidic chip was disposed of and the frame could be easily cleaned with solvents. The degassing through the lid of the perfusion frame enhanced the long-term stability of the system during experiments. Unavoidable temperature fluctuations of the cell-culture medium in the tubing between the pre-heated vials filled with medium and the chip lead to occasional formation of microbubbles. These bubbles tended to get trapped in low-flow regions within the microtissue compartments. Without the vacuum channels in the lid, these bubbles would have grown over time and would have displaced the spheroids or even pushed them out of their compartments. The vacuum lid successfully eliminated bubbles as the gases diffused through the PDMS layer.

During the experiments, the spheroids were continuously monitored by 2-photon imaging. In 2-photon microscopy, two intersecting beams of excitation light with large wavelengths (approx. twice that of the excitation wavelength) are used. Only in the focal area, where the two beams intersect, the photon flux is high enough to excite the fluorophores by 2-photon absorption. In comparison to other microscopy techniques, such as confocal imaging, 2-photon imaging features the following advantages: 1) The volume of excitation at the focal spot is small and limited to the imaging plane of interest, rather than illuminating all imaging planes along the *Z*-axis. Excitation is limited to the focal spot, which reduces phototoxicity and photobleaching and enables long-term observation of the spheroids. Furthermore, the image quality is increased due to reduced out-of-focus light (Rausch and Peker, 2020). 2) Large excitation wavelengths in the infrared range (800 nm) entail deep penetration of the IR waves in the spheroid. An analysis of the imaging quality in terms of resolution and optical penetration depth into the spheroids (Figure 2) revealed that cell nuclei were clearly detectable and distinguishable from each other throughout spheroids of at least 150 µm diameter. YZ- and XZ-projections of the acquired image stacks also illustrated the high resolution along the *Z*-axis. The temporal resolution of spheroid monitoring depended on the number of spheroids in the system and the number of Z-planes acquired per spheroid. For our experiments, 20 spheroids and 120 optical slices per spheroids were generally recorded, which resulted in a maximum temporal resolution of 85–95 min for continuous imaging. Selection of the desired imaging quality, the number of recorded Z-planes per spheroids, or the number of spheroids per treatment condition determine the temporal resolution of the experiment. At a total experimental duration of three days, we considered a resolution of 85 min sufficient.

The overall setup provides a high level of flexibility in terms of 1) number and nature of MTs per experimental condition, 2) duration and shape of PK profiles, 3) nature of intracellular fluorescent markers and selection of other optical readouts.

### Interpretation of Exposure-Depending Drug Effects

By combining a high-precision pumping system with a high-resolution imaging system, we could culture tumor spheroids under dynamic drug-exposure scenarios and monitor their drug responses in real-time. For the four different treatment conditions – PK-profile and constant-dose vehicle exposure, PK-profile and dose-matched, fixed-concentration exposure to BYL719 – we observed distinct dynamic changes in spheroid sizes and densities (Figure 4 to Figure 6).

The continuous increase in spheroid size in the vehicle control group indicated that the system was suitable for spheroid culturing purposes at constant perfusion rates. Even though the PK vehicle group featured slightly lower cross section spheroid areas than the constant-concentration vehicle group, the general growth behavior in terms of growth rates and total growth was very similar between the two control groups. In contrast, spheroids under constant-dose exposure to 9.3 µM BYL719 featured a size decrease, which was largest at the start of the exposure. The decreasing size of the tumor spheroids confirmed the previously reported sensitivity of the selected T47-D cell line to BYL719 (Elkabets et al., 2013). The extent of spheroid size decrease upon drug dosing was consistent with previously reported *in vivo* measurements of tumor size decreases in nude mice bearing BYL719-sensitive MCF7 cell-derived xenografts (Elkabets et al., 2013). The size changes of spheroids for a given treatment condition were reproducible between independent experiments, as evidenced by the similarity of the growth curves of individual spheroids (Figure 4) and the resulting standard deviations at specific measurement time points (Figures 5A,B). For this reason, both vehicle control conditions were evaluated together for effects at a cellular level. The number of neighboring nuclei – an indicator for the cell density within the spheroid – remained constant in the center and decreased in the shell region of the spheroids (Figure 6A). The initially lower and steadily decreasing density in the shell region can be attributed to an enlargement of cells, suggesting cell growth and proliferation in the outer spheroid region that is in direct contact with nutrient-rich medium. The treatment response of the spheroids did not depend on their position in the 10 consecutive compartments of each microfluidic channel (data not shown). This observation, together with the confirmation of the applied BYL719 concentration profiles at the chip outlet through mass spectrometry (Figure 3C), suggest that a flow rate of 10 μL min^−1^ through the device suffices to deliver the same dose to all spheroids in one channel. Downstream effects, such as drug metabolism-related drug depletion in the channel could, therefore, be excluded for our application. While this observation holds true for our choice of spheroid model and compound, special attention should be drawn on position-dependent behavior of spheroids when changing the cell model or substance. Two aspects that should be considered, in particular when operating the system at low flow rates or with more complex tissue models, are: 1) rapid metabolization rates of the compound – due to the metabolism of the spheroid or the compound properties – may continuously decrease the compound concentration and increase metabolite concentrations at downstream positions of the channel, and 2) secretory products of the tissues, such as cytokines or hormones, may affect downstream tissues.

Spheroids that were exposed to dynamic PK concentration profiles of BYL719 featured the same overall size decrease as that observed for spheroids under constant BYL719 exposure. Interestingly, the size decrease of the spheroids under PK exposure was not continuous, but these spheroids shrank and grew periodically. The control group under PK exposure to DMSO did not feature such an oscillatory growth pattern, which proved that the periodic size change of spheroids under PK exposure of BYL719 was not a consequence of changing vehicle concentrations or the perfusion setup/protocol. The periodic size change was caused by concentration fluctuations of the anticancer compound. A superposition of relative size changes during two subsequent PK exposure profiles and the respective BYL719 concentrations (Figure 5E) revealed that the size fluctuations inversely correlated with the BYL719 concentration changes. The initial BYL719-concentration peak in the PK profile was followed by a rapid decrease in spheroid size. Decreasing BYL719 concentrations towards the end of a dosing sequence coincided with a transition to spheroid growth. The same temporal characteristics were observed at single-cell level for the number of neighboring nuclei within a maximum distance of 20 µm to a reference nucleus. High BYL719 concentrations caused higher cell densities in both, the center and shell regions of spheroids under PK exposure (Figure 6C). In contrast, spheroids under constant exposure to 9.3 µM BYL719 featured a steadily increasing cell density in the center and shell regions (Figure 6B). The increasing cell density suggests cell shrinkage and can be explained by several potential processes: 1) The inhibition of the PI3K/AKT pathway by BYL719 might cause G0/G1 cell cycle arrest and/or decreased macromolecule production leading to cell shrinkage. Such an effect has previously been reported upon inhibition of mTORC1, a downstream node of the Pi3K/AKT pathway, by the anti-cancer drug Everolimus (Chen et al., 2019). 2) More generally, cell shrinkage can also represent a key hallmark of ongoing apoptosis (Bortner and Cidlowski, 1998). Considering the oscillating spheroid size and cell density in BYL719-treated spheroids during the first 48 h of treatment, the spheroid shrinkage could, at least in part, be attributed to cell shrinkage. Noteworthy, the presence of the observed change in cell density in the center and shell regions for both BYL719-treated conditions provides evidence for BYL719 penetrating into and affecting the entire spheroid. The transition from spheroid shrinkage to growth occurred at similar time points within the individual dosing sequences, e.g., 18.5 h after start of the first and 16 h after start of the second dosing sequence. This observation suggests that a treatment with BYL719 is most efficient when a dose above a certain threshold concentration is maintained. This hypothesis is supported by a pharmacological characterization of BYL719 published by Fritsch et al. (2014). Based on their *in vivo* study on the PK/PD/efficacy relationship of BYL719, they found that the plasma concentration of BYL719 must be maintained above the pathway-suppression IC_80_ for at least 45% of the dosing cycle to achieve tumor regression (Fritsch et al., 2014). By correlating the relative spheroid-size changes in our experiments with the BYL719 concentration profiles, the average BYL719 concentration, at which the size change switched from negative to positive was determined to 1.85 µM (1.3 μM at 18.5 h and 2.4 μM at 40 h). As there is a delay between the concentration change actuated by the pumping system and the manifestation of the antiproliferative effect of BYL719 in the tumor spheroid, it is difficult to precisely determine a threshold concentration from a measurement as displayed in Figure 5E.

We had a temporal resolution of 85 min, which is much shorter than commonly achieved in *vivo* experiments. The relatively short imaging intervals allowed us to track the short-term responses of tumor spheroids to realistic compound exposure scenarios. The obtained information on drug pharmacodynamics could potentially support the definition and fine-tuning of dosing regimens for cancer treatments. For example, our results showed tumor-spheroid growth towards the end of each PK exposure interval at low concentrations. Re-dosing at shorter intervals may help to maintain the plasma concentration of patients above an effective threshold concentration to avoid relapsing tumor growth. A clever dosing strategy can then be worked out without increasing the overall dose or without an unfavorable impact on the therapeutic index, in order to improve the therapy outcome.

### Added Value, Limitations, and Future Potential of the System

When comparing our system to other testing methods and systems for the investigation of PK/PD relationships, major advantages of our approach include 1) the potentially high temporal resolution, 2) the high spatial resolution through 2-photon microscopy, and 3) the experimental throughput in terms of numbers of replicates.

The most commonly used method to assess PK/PD relationships in preclinical studies during drug discovery are *in vivo* studies (Prantil-Baun et al., 2018). In such studies, disease-bearing mice are administered different doses of the active compounds over several days, during which absorption, distribution, metabolism, elimination (ADME) parameters are assessed and disease markers are monitored. For example, in the preclinical study for BYL719, conducted by Fritsch et al. (2014), tumor size in BYL719-treated mice was measured every 48 h over 9–16 days. While the overall outcome indicated a successful treatment at a daily dosage of 50 mg kg^−1^, the short-term growth dynamics of the tumor tissue between the 48 h measuring intervals remained unknown. The *in vivo* assessment of short-term dynamics through more frequent measurements of tumor size, however, is limited due to ethical and cost-related reasons that are associated with the use of animal model systems.

The increasing interest to assess PK-related drug effects *in vitro* has given rise to a growing list of platforms and approaches that focus on dynamic drug exposure of cellular model systems (Lohasz et al., 2019a; McAleer et al., 2019b; Guerrero et al., 2020; Komen et al., 2020). For example, Komen et al. (2020) developed a chip system for shear-stress-free, dynamic compound exposure of 2D cell layers. The authors could show that Oxaliplatin efficacy is mostly determined by continuous exposure times and not by peak concentrations. Guerrero et al. (2020) presented a chip system that includes an on-chip mixing structure and a culture compartment that is suitable for 2D and hydrogel-based 3D cell-culture approaches. With their system, they assessed how the treatment schedule of combinatorial anti-cancer treatment with the commonly used drugs doxorubicin and gemcitabine influenced treatment efficacy. Both exemplary studies demonstrated the benefit of *in-vitro* chip systems for assessing exposure-dependent treatment efficacy. An accurate assessment of PK/PD relationships *in vitro* could enable the optimization of treatment plans before testing compounds in animals or administration to participants in clinical trials. However, a potential shortcoming of many studies is that they rely on endpoint analyses after 48–72 h of drug treatment and neglected short-term responses of target tissues. Moreover, the experimental throughput is often limited through the need of a pump unit for each individual culturing compartment.

In contrast, the system proposed here features a comparably high temporal resolution and throughput through combination of a perfusion/culturing platform with a fully automated imaging system. We use injection-molded disposable polystyrene chips in a perfusion frame; every chip features two channels, each of which has 10 culturing compartments, so that up to 10 spheroids can be simultaneously exposed to a certain dosing regimen. The current system offers four channels with 40 spheroids that can be perfused by using eight pressure pumps and flow rate sensors. Moreover, the disposable polystyrene-based chip is clamped onto the re-usable perfusion frame by a simple mechanism. Chips loaded with microtissues can be exchanged between subsequent experimental runs without complete re-assembly of the microfluidic tubing.

While we have increased experimental throughput compared to published systems, pump-driven microfluidic systems are not the best choice for high-throughput experiments, as a large number of pieces of equipment, such as pumps, sensors, reservoirs and tubing are required. The corresponding experiments then become increasingly expensive and complex. Furthermore, connections to external pumps and tubing are, in most cases, not compatible with highly automated environments. There is a number of alternative perfusion approaches to overcome the use of complex and expensive precision equipment by exploiting gravity (Trietsch et al., 2013; Oleaga et al., 2016; Busche et al., 2020), centrifugal forces (Gorkin et al., 2010; Schneider et al., 2020), or capillary forces (Zimmermann et al., 2007; Olanrewaju et al., 2018). However, the corresponding devices cannot provide similarly precise concentration and flow control as pump-driven systems (Lee et al., 2016; Lohasz et al., 2019a; McAleer et al., 2019b). In our system, we used a single-pass perfusion scheme that, in contrast to recirculating perfusion systems, washes out and prevents the enrichment of drug metabolites or cellular signaling molecules in the liquid phase. Such single-pass perfusion systems limit their use to substances with a direct functional impact. Prodrugs, for example, need to be transformed to active metabolites to produce the desired drug effect, and a threshold concentration needs to be reached that then triggers the desired response in the target organ (Lohasz et al., 2020; Rajan et al., 2020). In a system, in which metabolites or other slowly generated molecules are continuously flushed out, drug effects may remain undetected. The same holds true for secondary drug effects through secreted cellular signaling molecules. Therefore, the perfusion pattern (e.g., single-pass or recirculating) has to be carefully chosen considering the mode of action of the drug (e.g., direct or indirect through metabolites or secondary effects) to obtain meaningful results in drug efficacy and/or toxicity testing. Here, we used BYL719, which is not a pro-drug, and for which it is assumed that secreted signaling molecules play a minor role if any in suppressing tumor growth. Therefore, a single-pass pattern was a suitable option.

An essential part of PK/PD relationship characterization in drug discovery is to define a so-called therapeutic window, i.e., a drug concentration range, within which the compounds have the desired effect without showing significant adverse events (Katzung, 2018). The therapeutic windows for many commonly used anticancer medications are particularly narrow so that the definition of doses and dosages with clinical efficacy and without severe side effects for treatment of patients is challenging (Fendt, 2017; Luengo et al., 2017). Our chip system could be extended to a multi-tissue format for simultaneous testing of drug efficacy on the target tissue and potential toxicity effects on other tissue types. While different organ models have been combined for parallel efficacy/toxicity testing by other groups (McAleer et al., 2019a; Rajan et al., 2020; Schimek et al., 2020; Skardal et al., 2020; Weng et al., 2020), only few systems feature the possibility to apply PK concentration profiles (McAleer et al., 2019b; Herland et al., 2020). Simultaneous efficacy/toxicity testing helps to compare and define maximum concentrations and dosing profiles and intervals that represent the best trade-off between treatment efficacy and toxicity in order to optimize the treatment outcome while ensuring patient safety. Especially the use of primary cells or material from patient biopsies could increase the relevance of the experimental results and allow for assessing patient-specific responses to different treatment regimens. The use of our system for such simultaneous efficacy/toxicity testing, however, will require additional endpoints to assess and distinguish the responses of different tissue models to a compound. In addition to the 2-photon imaging for size and morphology assessment, the automated outlet sampling system (Supplementary Figure S2) can be used to sample the perfusate at distinct time points. The quantification of organ-specific toxicity markers (e.g., aspartate transaminase/alanine transaminase for liver tissue, or Troponin-I for cardiac tissue) will then provide time-dependent information on organ toxicity. Furthermore, all spheroids can potentially be harvested from the system for downstream analyses, such as biochemical viability measurements or the quantification of tissue-resident toxicity markers.

Concerning our choice of cell model, it is widely accepted that 3D-cultured cancer models outperform traditional 2D cell culturing approaches in terms of representative morphology, architecture, and substance gradients across the tissue (e.g., oxygen, glucose, chemokines, etc.). Nevertheless, some aspects (e.g., tumor heterogeneity, presence of vasculature or a lymphatic system, etc.) are not recapitulated in 3D spheroids. To what extent 3D tumor spheroids may be representative for tumors in patients, the limitations of 3D cell-culture models and their usefulness for cancer research have been discussed in detail elsewhere (Jensen and Teng, 2020; Pinto et al., 2020).

While we successfully employed our microfluidic platform to monitor dynamic drug effects in different regions of the spheroids, the utilized 2-photon microscopy also offers the potential to measure specific target effects. Through use of specific fluorescent cellular labels or distinct reporters, mechanistic studies of the relationship between drug application and target effects can be performed.

Given the above-mentioned capabilities, the microfluidic chip system holds great potential for implementation into the drug discovery and development pipeline in order to expedite *in vivo* studies. In more detail, the system can be employed to prioritize advanced compounds during lead optimization 1) by comparing the 2D/3D static *in vitro* efficacy to different PK exposure profiles, and 2) by addressing mechanistic questions around what kind of exposure profile (c_max_, AUC, or time-above-threshold) is required to optimally drive efficacy for a given target class. Preselected concentration profiles featuring high *in vitro* efficacy can subsequently be tested at a more systemic level *in vivo* by adjusting the dosing regimen of an existing compound or by initiating the synthesis of new compounds with desired PK properties. Applying such an approach prior to lengthy efficacy studies will help to reduce animal usage and shorten development timelines.

## Conclusion

In conclusion, we developed a versatile microfluidic tissue-culturing platform for exposure of 3D cellular models to dynamically changing drug-concentration profiles and for continuous monitoring of the pharmacodynamic effects by means of 2-photon microscopy. As a proof-of-concept, we showed how the efficacy of an antiproliferative drug may differ between constant-dose exposures schemes that are often applied *in vitro* and cyclic PK exposure profiles resulting from the ADME characteristics of a compound. We used the system to expose T47-D breast cancer spheroids to realistic pharmacokinetic drug concentration profiles of the recently approved anti-cancer compound BYL719 over a total duration of 3 days. The spatial and temporal resolution of our system allowed for the detection of tumor spheroid growth dynamics at tissue and single-cell level, which inversely correlated with drug concentrations. The system offers great flexibility with respect to useable microtissue models and dosing scenarios and provides a versatile tool for in-depth preclinical evaluation of drug efficacy. Moreover, it can be used for monitoring toxicity and target effects.

## Data Availability

The raw data supporting the conclusions of this article will be made available by the authors, without undue reservation.
