# Supplementary material for: A Microphysiological Cell-Culturing System for Pharmacokinetic Drug Exposure and High-Resolution Imaging of Arrays of 3D Microtissues
Source: Front Pharmacol. 2021 Dec 21;12:785851. doi: 10.3389/fphar.2021.785851 (PMC8954798; doi:10.3389/fphar.2021.785851)
Supplement: Supplementary file 1 [file DataSheet1.PDF]

Supplementary Material

1 Supplementary Materials and Methods

1.1 Microfluidic device fabrication

The microfluidic chip system was fabricated by modifying and expanding a commercially available microfluidic microtissue-culturing chip (Akura^{TM} Flow, InSphero AG, Schlieren, Switzerland). The injection-molded polystyrene chip has the size of a microscopy slide (2.5 mm x 7.5 mm) and features two independent microfluidic channels, each of which interconnects ten microtissue (MT) compartments (Lohasz et al.). The chip was originally developed for microtissue culturing applications using gravity-driven perfusion. For this project, the chip was modified to allow for connection with externally controlled pressure pumps to realize perfusion.

The chip was obtained unsealed with exposed channel structures. Computer numerical controlled (CNC) micromilling was then used for the following chip modifications: (i) at the bottom, the most narrow sections of the flow path, which were located at the intersection of the medium reservoirs and the microfluidic culturing channel, were enlarged to decrease the hydraulic resistance; (ii) at the top, the wall structures around the access ports to the MT compartments were removed to enable closure of the MT compartments to avoid medium leakage. Flat-end milling heads with diameters of 0.5 mm, 1 mm and 1.5 mm (DIXI Polytool S.A., Le Locle, Switzerland) were used for these modifications. After these modifications by CNC micromilling, the channel structures at the bottom of the chip were sealed with custom-cut, pressure-sensitive adhesive film (Brooks Life Sciences, Chelmsford, MA, USA). All internal channel surfaces were coated with a hydrophilic, non-adhesive coating, provided by InSphero AG, to prevent attachment of the spheroids during experimentation. All modifications performed to the chip are illustrated in Supplementary Figure 1.

For interfacing the modified chips with pressure pumps, a dedicated aluminum frame was CNC machined. The frame featured two recesses for the simultaneous experimentation with two chips. The inlet and outlet reservoirs of the chips were closed with custom-made polyether-ether-ketone (PEEK) clamps. Water-jet-cut silicone sealing prevented leakage of perfusion medium between the clamps and the reservoir rims. Extrusions at the bottom side of the PEEK clamps minimized excessive dead volumes of medium within the reservoirs of the chip. At the top side of the clamps, threaded holes allowed for connection of the microfluidic tubing with SuperFlangeless Fitting Nuts (Kinesis GmbH, Langenfeld, Germany). For compatibility with standard microscopy stages, the assembled aluminum frame featured the same footprint as standard microtiter plates (ANSI/SLAS).

A lid was fabricated to tightly seal the access ports to the MT compartments. Therefore, multiple layers were laser-cut from acrylic sheets using a Universal Laser Systems VLS 2.30 laser cutter (Ferutec AG, Bubikon, Switzerland). The layers were then aligned and assembled with double-sided sticky tape. The lid featured internal channel structures. Openings at the bottom side of the lid aligned with the MT compartments of individual microfluidic channels. At the top of the lid, a barbed tube adapter was attached onto the opening to the channel structures. To close the MT compartments, 1 mm thick polydimethylsiloxane (PDMS) layers were used to separate the microfluidic chip from the acrylic lid. The lid was then pressed onto the chips by screwing it directly onto the aluminum frame. A vacuum tube was attached to the barbed tube connector on the lid. Applying a vacuum

within the channel structures of the lid provided chip degassing and prevented the formation of bubbles throughout an experiment. The semi-permeable PDMS sheet separating the chips from the lid allowed air and gas bubbles to pass while liquids remained within the microfluidic channels of the chip. The details of the chip degassing were previously described (Huang et al., 2020).

1.2 Computational modelling

The dynamics of substance concentrations within the MT compartment upon a stepwise compound concentration increase through perfusion was numerically modelled using the COMSOL Multiphysics® software (COMSOL AB, Stockholm, Sweden). A half-section of the 3D model of the microfluidic chip, which included a single MT compartment, was imported into COMSOL. A spheroid with a diameter of 250 µm was placed in the center of the MT compartment.

For the fluid dynamics model, the viscosity and density of the cell-culture medium at 37 °C were set to that of water (i.e., 6.91×10^{-4} Pa s and 993.3 kg m^{-3}). No-slip boundary conditions were assumed at all liquid-channel and liquid-spheroid interfaces. A symmetry boundary condition was set on the mid-plane going through the height of the MT compartment (Supplementary Figure 5A). A constant laminar inflow of 10 µL min^{-1} was set at the inlet and a null pressure was set at the outlet.

For the substance transport model, diffusive and advective changes in the MT compartment were modeled. For the advective transport component the previously attained solution of the fluid dynamics model was used. As the diffusion coefficient of BYL719 was unknown, it was estimated based on the relationship between the molecular weight and the hydrodynamic radius (Stoke's radius) of molecules, which was built on data taken from Armstrong et al. (2004). With a molecular weight of 441.5 g mol^{-1}, the hydrodynamic radius of BYL719 was estimated to be 0.49 nm. Using the Stokes-Einstein equation, the diffusion coefficient of BYL719 in water at 37 °C was estimated to 6.71×10^{-10} m^2 s^{-1}. The initial conditions were defined with a null concentration of the compound throughout the medium and a constant influx of the generic substance at a relative concentration of 1 at the inlet. The relative concentration was plotted on the colinear planes through the MT compartment height and through the channel width (as indicated in Supplementary Figures 5A and 5C).

1.3 References

ANSI/SLAS (2004). "ANSI/SLAS 1-2004: Microplates - Footprint Dimensions".).

Armstrong, J.K., Wenby, R.B., Meiselman, H.J., and Fisher, T.C. (2004). The hydrodynamic radii of macromolecules and their effect on red blood cell aggregation. *Biophysical journal* 87(6)**,** 4259-4270. doi: 10.1529/biophysj.104.047746.

Huang, C., Wippold, J.A., Stratis-Cullum, D., and Han, A. (2020). Eliminating air bubble in microfluidic systems utilizing integrated in-line sloped microstructures. *Biomed Microdevices* 22(4)**,** 76. doi: 10.1007/s10544-020-00529-w.

Lohasz, C., Rousset, N., Renggli, K., Hierlemann, A., and Frey, O. (2019). Scalable Microfluidic Platform for Flexible Configuration of and Experiments with Microtissue Multiorgan Models. *SLAS Technol* 24(1)**,** 79-95. doi: 10.1177/2472630318802582.

2 Supplementary Figures

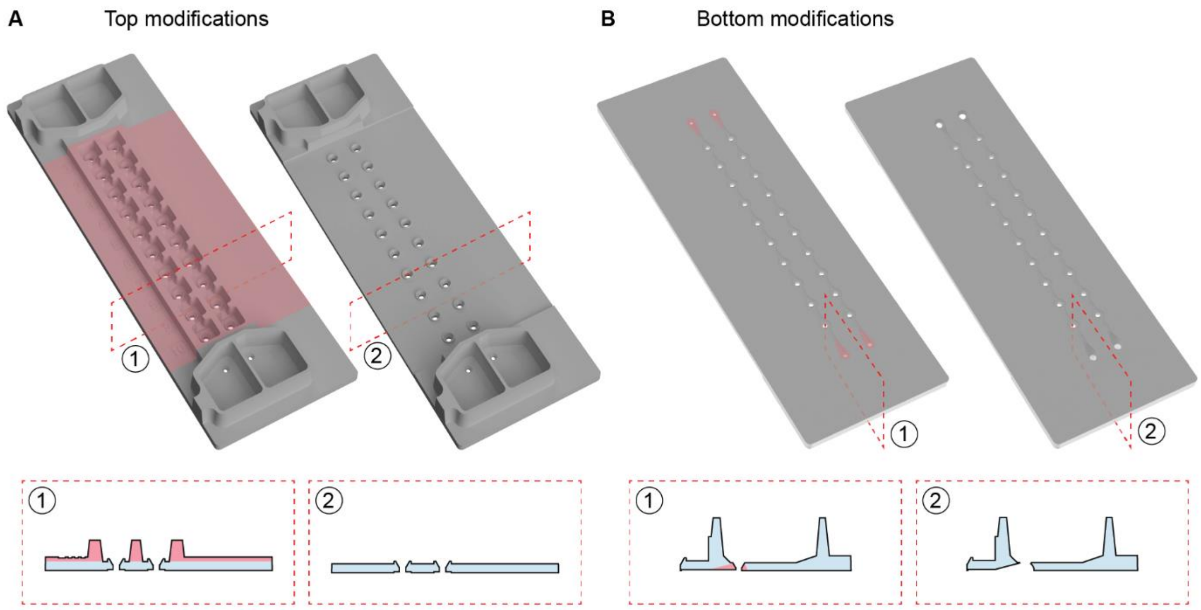

Supplementary Figure 1: Modifications of the injection-molded polystyrene chip that were performed by CNC micromilling. (A) Wall structures that separated the individual microtissue loading ports were removed at the top side. (B) The intersections between the microfluidic channel and the reservoirs were enlarged at the bottom of the chip. The chip is displayed before (left) and after (right) the CNC micromilling process. The modified (removed) regions are indicated in red in the chip images and the cross sections below the images.

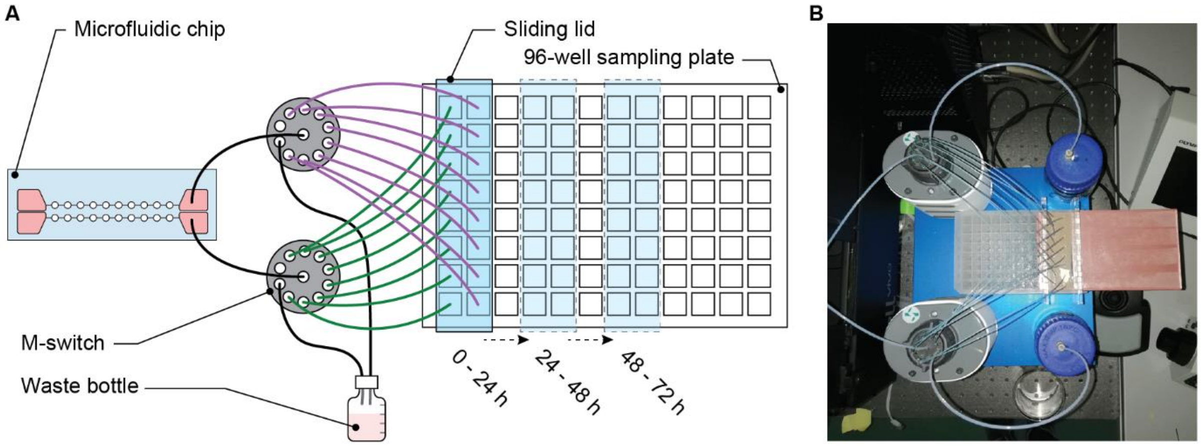

Supplementary Figure 2: Downstream sampling unit to collect fractions of the perfusate for BYL719 quantification at distinct time points of the pharmacokinetic drug-dosing protocol. (**A**) Schematic drawing of the setup. The setup consisted of M-switches (one per channel) and a 96-well sampling plate (well volume = 2 mL) with a custom-made sliding lid for the attachment of microfluidic tubing. FEP tubing (ID = 250 µM) was used connect the chip outlets to the M-switches and waste bottles. PEEK tubing (ID = 125 µm) was used between the M-switches and the sampling plate (purple and green colors are used to distinguish the two different channels). For sampling, the M-switches sequentially directed the medium to distinct positions on the 96-well sampling plate. During breaks between the sampling times, the medium was directed into a waste bottle. For each PK profile, eight samples were taken at different time points. The sliding lid was manually moved to empty positions on the 96-well plate every 24 h. (**B**) image of the sampling setup.

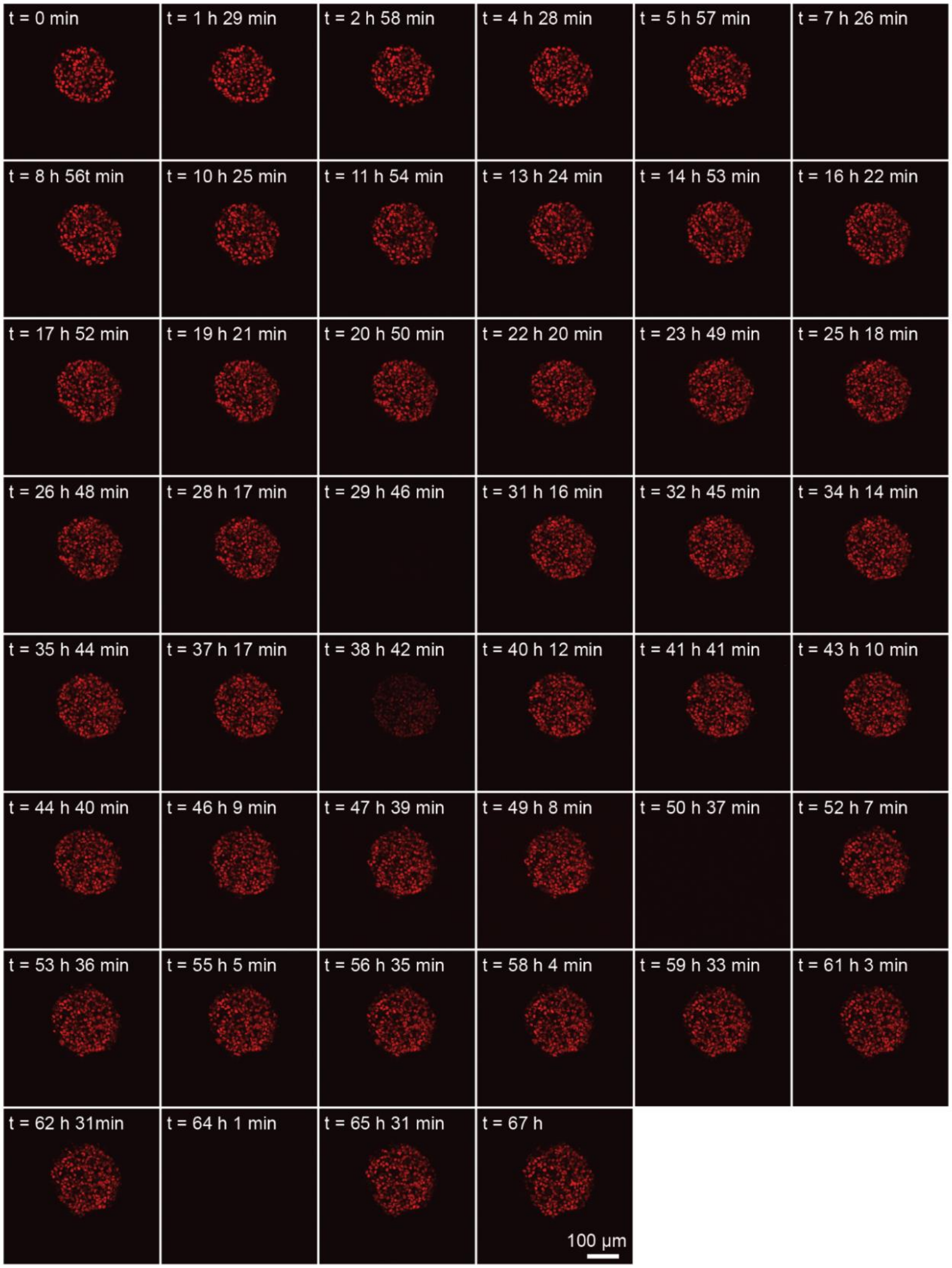

Supplementary Figure 3: Representative time series of a NucLight Red-labeled T47-D tumor spheroid in perfusion culture under BYL719 dosage at a constant concentration of 9.3 µM. Images are displaying the maximum intensity projection of a stack with 121 slices for each imaging time point. Images without fluorescence signal (t = 7 h 26 min, t = 29 h 46 min, t = 50 h 37 min, t = 64 h 1 min) display occasional events, at which a bubble within the perfused lens immersion system of the microscope temporarily interfered with the image acquisition.

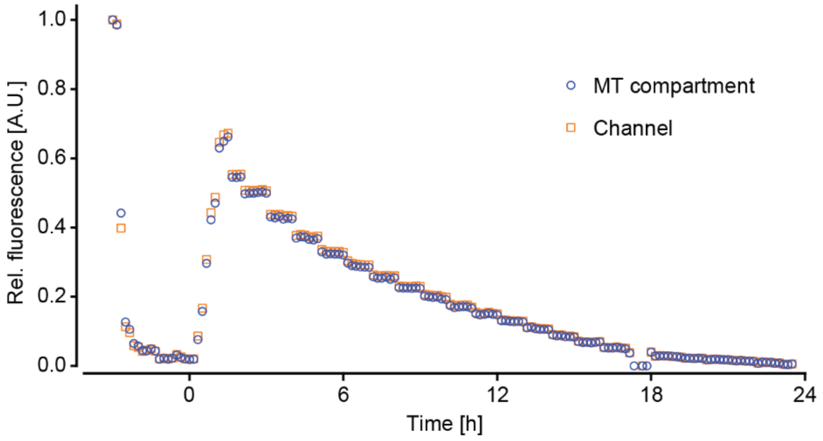

Supplementary Figure 4: Evaluation and comparison of the normalized fluorescence signal inside the culturing compartment and in the perfusion channel. The applied concentration profile included an initial sequence of 30 min at 100% dye concentration and 2.5 h at 0% dye concentration before starting the modeled PK profile for BYL719 over 24 h.

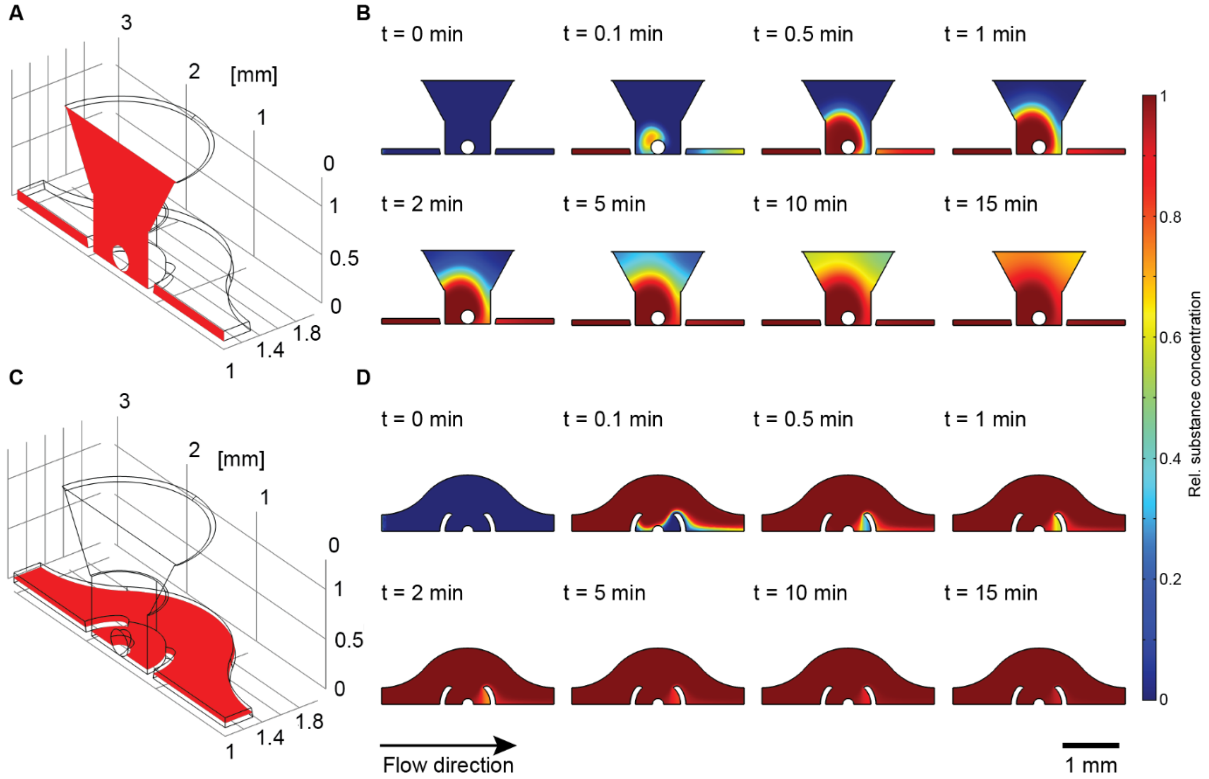

Supplementary Figure 5: Modeling of the relative concentration change over time within a MT compartment upon a stepwise concentration increase by perfusion. Illustration of the cross sections through **(A)** the MT compartment height and **(C)** through the channel width to display the changing concentrations. **(B** and **D)** The relative concentrations along the previously defined cross sections was plotted at different time points ranging from 0 to 15 min.
